# Supplementary material for: CMDdemux: an efficient single cell demultiplexing method
Source: Nucleic Acids Res. 2026 Apr 25;54(8):gkag360. doi: 10.1093/nar/gkag360 (PMC13109729; doi:10.1093/nar/gkag360)
Supplement: gkag360_Supplemental_File [file gkag360_supplemental_file.pdf]

Supplementary Materials

Supplementary Methods

1 Global CLR normalization

Global CLR normalization refers to normalizing HTO counts directly across cells. The global CLR-normalized value is defined as:

$$h'_{i,c} = log \frac{HTO_{i,c}}{(\prod_{c=1}^{n} HTO_{i,c})^{\frac{1}{n}}}$$

where n is the total number of cells.

2 Determining the number of clusters

For ideal clustering, each hashtag is expected to have a single cluster with high expression compared to all other clusters. Since eight hashtags were used to label the samples in the PBMC dataset, we initially set $k = 8$ for clustering (Fig. S1). All hashtags exhibit a clear high expression pattern in one cluster, except for HTO-G, which shows only moderate expression in cluster 1. We then tested $k = 9$ by adding one more cluster. It became clear that HTO-G shows higher expression in cluster 7 when using $k = 9$. The highest local CLR value for each hashtag and its corresponding cluster is summarized in Table S1. As shown in the table, the expression of HTO-G increases by 1.07 when applying 9 clusters compared to 8 clusters, which exceeds the default threshold of 1. Therefore, choosing 9 clusters is preferable, as it results in a higher expression of HTO-G in a single cluster while maintaining similar expression patterns for other hashtags compared to the 8-cluster solution.

3 Solving conflicting hashtags

Using the average expression-based method, multiple hashtags may be assigned to the same cluster in rare cases. This indicates that the cluster expresses multiple hashtags at high levels, as reflected by high median local CLR values across those hashtags. When this occurs, we first exclude non-core cells and then calculate the median and interquartile range (IQR) of local CLR values for each conflicting hashtag across within each cluster. For each conflicting hashtag i, we identify the top two clusters, l_1 and l_2,

with the highest median local CLR values. We then compute a normalized difference score D as follows:

$$D_i = \frac{|Med(h_{i,l_1}) - Med(h_{i,l_2})|}{1/2(IQR(h_{i,l_1}) + IQR(h_{i,l_2}))}$$

The conflicting cluster is then assigned to the hashtag with the highest D value, reflecting the most distinguishable expression. For any remaining conflicting hashtags, each will be assigned to the cluster (among the unlabelled ones) with the highest median local CLR value.

For example, suppose there are four HTOs, and the clusters are initially labelled such that HTO_1, HTO_2, HTO_3, and HTO_4 are assigned to clusters 1, 1, 2, and 4, respectively. In this case, HTO_1 and HTO_2 are conflicting hashtags, both assigned to cluster 1. For HTO_1, the top two clusters by median local CLR value are clusters 1 and 2; for HTO_2, they are clusters 1 and 3. We then compute D_{HTO_1} and D_{HTO_2} based on these pairs. If $D_{HTO\_1} = 0.2$ and $D_{HTO\_2} = 0.1$, cluster 1 will be assigned to HTO_1, as it shows a more distinct separation. The remaining unlabelled cluster 3 will then be assigned to HTO_2.

4 Rescuing droplets with unlabelled hashtags

We first examine whether any unlabelled hashtags are present in the data. For each hashtag, the median local CLR value across clusters is calculated. Theoretically, an unlabelled hashtag should exhibit low expression across all clusters. To identify such cases, we select the top two clusters with the highest local CLR values for each hashtag and set a default threshold of 0.5. If the difference in median local CLR between the top two clusters is less than this threshold, the hashtag is considered unlabelled. An example from the EMBRYO LMO dataset is shown in Fig. S2(a). Except for MULTI_2, all hashtags show a uniquely high expression in one cluster. For MULTI_2, however, the median local CLR values across all clusters range between -2 and -4. The top two clusters for MULTI_2 are cluster 1 and cluster 5, with median local CLR values of -2.29 and -2.56, respectively. The difference between these two clusters is 0.27, which is smaller than the default threshold of 0.5. Therefore, MULTI_2 is classified as an unlabelled hashtag.

After identifying the unlabelled hashtags, the next step is to rescue droplets associated with these hashtags from the group of negatives. First, droplets classified either as unlabelled hashtags or as negatives are grouped together as temporary negatives. For each temporary negative droplet, we calculate the difference (D) between the local CLR value of the unlabelled hashtag (u) and the maximum local CLR value among all hashtags, defined as:

$$D_c = h_{u,c} - max_{i=1,...,N}(h_{i,c})$$

where $h_{u,c}$ is the local CLR value of hashtag u in cell c.

The log expression value of the unlabelled hashtag, $log(HTO_{u,c})$, is also recorded. In theory, droplets with a CLR difference (D_c) close to zero and a high $log(HTO_{u,c})$ value are likely true singlets belonging to the unlabelled hashtag. An example of rescuing MULTI_2 singlets from the EMBRYO LMO dataset is shown in Fig. S2(b). The scatter plot is divided into four regions; true MULTI_2 singlets appear in the top-right region, where droplets have both high MULTI_2 expression and a CLR difference close to zero. As a result, six temporary negatives were rescued and reclassified as MULTI_2 singlets.

5 Reclassifying droplets in the negative clusters

In this paper, we discussed the presence of extra cluster(s) in the data, such as in the EMBRYO MULTI-Seq CMO dataset and the PBMC dataset. Although the majority of droplets in these extra clusters are expected to be negatives, some singlets and doublets may still be present. To improve classification, we reclassify droplets within these negative clusters based on their gene expression profiles. Specifically, two thresholds are established to identify potential doublets: the 0.9 quantile of mRNA library size across all cells, and the median mRNA library size of all doublets identified during the initial demultiplexing. Droplets in the negative clusters with mRNA library sizes exceeding either threshold are reclassified as doublets. We also reclassify potential singlets within the negative clusters. For this, the threshold is set as the median mRNA library size of each singlet cluster. Based on the minimum Mahalanobis distance of each droplet, the corresponding singlet cluster and its threshold are determined. Droplets in the negative clusters with mRNA library sizes larger than their corresponding singlet thresholds are reclassified as singlets.

Supplementary Tables

Table S1: **Maximum local CLR value across different values of k in PBMC data.**

Hashtag	K=8		K=9		Difference of maximum local CLR between different k
	Cluster	Maximum local CLR	Cluster	Maximum local CLR	
HTO-A	6	3.33	6	3.35	0.02
HTO-B	7	3.75	8	3.76	0.01
HTO-C	2	3.44	2	3.45	0.01
HTO-D	8	4.16	9	4.16	0
HTO-E	4	3.79	4	3.8	0.01
HTO-F	3	3.87	3	3.89	0.02
HTO-G	1	2.81	7	3.88	1.07
HTO-H	5	3.69	5	3.7	0.01

The table shows the maximum local CLR value for each hashtag and its corresponding cluster. The last column represents the difference between the maximum local CLR values for $k = 9$ and $k = 8$.

Table S2: **Summary of demultiplexing methods.**

**Method**	**Year**	**Basic Model**	**Description**	**Limitations**
HTODemux[1]	2018	Negative binomial	1. Normalize the HTO counts using across-cell CLR normalization.<br>2. Perform K-medoids clustering on the normalized HTO values.<br>3. Exclude the cells with extreme HTO values.<br>4. Fit the HTO counts from the remaining cells to a negative binomial distribution.<br>5. Set up a threshold based on a quantile of the distribution.<br>6. Cells with HTO lower than the threshold are "negative", whereas those higher than the threshold are "positive". Cells assigned to exactly one "positive" hashtag are singlets, and those assigned to more than one "positive" hashtag are multiplets.	HTODemux assigns too many negatives in low-quality data. This is because it has a conservative threshold to define positive cells. HTODemux defines one more k-medoids cluster as a negative cluster. This works well on the example PBMC dataset provided in the paper. However, negative droplets do not always cluster together in the real world. For example, if a high-quality dataset only has a limited number of negative droplets, such as $<1\%$, then those negatives will scatter everywhere in the major singlet clusters instead of forming a negative cluster. Another example is when all droplets have low cell hash counts. Then cells tend to form a giant cluster without separating into clear clusters. In the above two cases, a compulsory number of clusters does not make sense. In the paper, the author mentioned "We identified the k-medoids cluster with the highest average HTO expression and excluded these cells." But if we check the code, they actually exclude the droplets with lowest average HTO expression,

Method	Year	Basic Model	Description	Limitations
				which are negatives, to set up the threshold for positive and negative cells. Assuming that the dataset does not have a negative cluster, some genuine singlets will be clustered as negatives by HTODemux, and they will be excluded when defining the threshold for positives and negatives, so that the threshold for the positive droplets will be higher than expected, and more droplets will be defined as negatives. That is the reason why this clustering strategy will result in a conservative cut-off and assign more droplets as negatives by HTODemux.
hashedDrops[2]	2019	Log fold change	1. Determine the empty droplets.<br>2. Estimate the ambient contamination and adjust the HTO counts by the scaled ambient profile.<br>3. Singlets are defined by the log fold change between the most abundant HTO and the second most abundant HTO. The default cut-off is 3 median absolute deviations (MAD).<br>4. Doublets are defined by the log fold change between the second most abundant HTO and the ambient contamination. The default cut-off is 3 MADs.	hashedDrops often assigns more negatives than other methods. It is possible that the cut-off of log fold change is too conservative. If the algorithm uses a loose cut-off, then more negative droplets will possibly be assigned as singlets.

Method	Year	Basic Model	Description	Limitations
DemuxEM[3]	2019	Expectation-maximization (EM)	1. The probability model includes the proportion of HTO unique molecular identifiers (UMI) coming from the background and the true signal.<br>2. The EM algorithm is used to estimate the proportion of hash UMIs originating from the background distribution. The true signal is then inferred by subtracting the estimated background component.<br>3. If the estimated hashtag UMI count is <10, the cell is called "unassigned". If only one sample has at least 10% of HTO UMIs, the cell is assigned to be a singlet. Otherwise, the cell is a multiplet.	DemuxEM is unable to output results when applied to most low-quality data. It is unclear whether the issue lies in the model itself or its implementation. The model assumes that hash UMIs originate from two components—background and signal—and estimates the background distribution using k-means clustering with k=2. The cluster with lower UMI expression is treated as the negative (background) component. However, in low-quality data, this two-component assumption may not hold. As a result, the k-means clustering used to separate negative and positive components may perform poorly.

Method	Year	Basic Model	Description	Limitations
GMM-Demux[4]	2020	Gaussian mixture	1. Normalize the HTO counts using across-cell CLR normalization.<br>2. Fit the CLR normalized HTO values into a two-component Gaussian mixture model.<br>3. Use Bayes' posterior probability to classify each cell as belonging to hashtag-free gel beads or hashtag-enclosing gel beads.<br>4. Use the mutually exclusive rule for the probabilities to assign a cell to be a multiplet.	The two-component Gaussian mixture works well on high-quality data due to its inherent ability to capture two modalities: background noise with a lower mean and true signal with a higher mean. However, low-quality data deviates from this bimodal distribution, causing GMM-Demux to misclassify low-quality hashes as either doublets or negatives. Consequently, it may fail to retain true singlets in low-quality data. Additionally, in their original paper, the authors acknowledge that they cannot resolve the issue of data with low hash UMIs or droplets with ambiguous probabilities.
$\mathrm{BFF}_{\mathrm{raw}}$[5]	2022	Bimodal log HTO count	1. Fit the log (HTO count + 1) values into a smooth density curve by using kernel density estimation.<br>2. The threshold is determined by the minimum density of the fitted bimodal distribution.<br>3. Cells below the threshold are defined as negatives, whereas others are positives. Cells that are positive for exactly one hashtag are singlets, while those positive for multiple hashtags are doublets.	The BFF methods do not work well on low-quality data. The underlying assumption of BFF methods is that the HTO count is bimodally distributed, allowing thresholds to be set to distinguish noise from the true signal. However, low-quality data generally does not follow this pattern, making BFF methods ineffective in classifying poorly hashed droplets. Additionally, although BQN ensures that all hashes

Method	Year	Basic Model	Description	Limitations
$BFF_{cluster}$[5]	2022	Bimodal quantile normalization (BQN)	1. Normalize the HTO counts using BQN, which first separates cells into positive and negative groups for each hashtag using a threshold, and then applies quantile normalization to each group separately. The cut-off for positive and negative cells is determined by BFF_{raw}. Then combine the quantile-normalized negative and positive cells.<br>2. Set up a threshold for the highest BQN HTO distribution across cells. If the highest BQN is below the threshold, the droplet is assigned to be negative.<br>3. Set up a threshold for the second highest BQN HTO distribution across cells. If the cell's second highest BQN is larger than the threshold, the cell is assigned to be a doublet.<br>4. Compare the differences between the cell's highest and second highest BQN value. If less than a threshold, it will be assigned to be a singlet. Otherwise, it will be assigned to be a doublet.	are distributed consistently, this normalization method removes individual features of each hash. Furthermore, BFF methods heavily rely on threshold selection. Whether BQN can provide a reliable threshold for each hash remains uncertain.

Method	Year	Basic Model	Description	Limitations
demuxmix[6]	2023	Negative binomial regression mixture model	1. Fit a negative binomial regression model between the expected HTO count and the number of detected genes in the RNA expression data.<br>2. Use the EM algorithm to estimate parameters, with initial values derived from k-means clustering, which defines positive and negative droplets.	Demuxmix assigned fewer singlets compared to other methods in low-quality data. This is possibly because cell hashing and gene expression profiles come from two different modalities. The linear relationship between HTO count and the number of detected genes does not hold in low-quality data. Moreover, this linear relationship applies separately to each cell type[7], meaning that complex heterogeneous cell types can negatively impact the model.
deMULTIplex2[7]	2024	GLM-NB (Negative binomial generalized linear model)	1. Using GLM-NB fit the relationship between the contaminating tag count and total tag count. Using a second GLM-NB fit the relationship between the contaminating multi-tags and total tag count to model the positive labelled tag.<br>2. The parameters of the model is estimated based on EM algorithm with cosine similarity with canonical vector of each tag.	deMULTIplex2 does not work well for data with small counts. This is possibly because small hashing counts do not fit the GLM-NB model well, which may lead to unreliably fitted parameters. Additionally, some datasets with small counts fail to converge in the EM algorithm.

Table S3: **Summary of data used for benchmarking.**

Data	Quality	Number of hashtags	Number of cells	Technology	Tissue/Cell type	Ground truth	Gene expression data	Low-quality type	Publication	Data source
Human brain	High-quality	8	2,754	Single nucleus hashing	Human brain cortex	demuxlet	✓	NA	Gaublomme et al., 2019[3]	`https://hub.docker.com/r/regevlab/demuxem`
BAL		8	24,804	TotalSeq antibodies	Paediatric bronchoalveolar lavage fluid	Vireo	×		Howitt et al., 2023[8]	`https://zenodo.org/records/8304003`
Vehicle mouse		3	7,403	TotalSeq antibodies	Vehicle treated mouse immune cells	×	✓		Virassamy et al., 2023[9]	`https://zenodo.org/records/7325111`
Treated mouse	Low-quality	3	4,436		Ipatasertib treated mouse immune cells		×(Available upon requests)	Uneven labelling with weak signals		`https://zenodo.org/records/16976382`
OT		4	9,405	TotalSeq antibodies	Ovarian carcinoma	Vireo	✓	Uneven labelling with strong signals	Hippen et al., 2023[10]	GSE217517
EMBRYO MULTI-Seq LMO		12	9,745	MULTI-Seq lipid modified oligo (LMO)	Mouse E18.5 brain	×	✓	Uneven labelling with unlabelled hash	Brown et al., 2024[11]	`https://zenodo.org/records/11134439`
EMBRYO MULTI-Seq CMO		12	19,494	Custom MULTI-Seq cholesterol modified oligo (CMO)				Contamination		
PDX CellPlex		4	4,327	CellPlex	Nuclei from human ovarian carcinosarcoma patient-derived xenograft (PDX)	×	✓	Uneven labelling with strong signals / Low-input		
PDX Hashtag Ab		4	7,071	TotalSeq antibodies				Low-input		
PDX MULTI-Seq CMO		4	4,178	Custom MULTI-Seq CMO				Uneven labelling with strong signals / Low-input		
PBMC		8	21,522	Antibodies	Peripheral blood mononuclear cells (PBMC)	×	✓	Empty droplets	Stoeckius et al., 2018[1]	GSE108313

Table S4: **Influence of tuning parameters.**

**Data**	**CMDdemux**	**HTODemux**	**deMULTIplex2**	**demuxEM**	**demuxmix**	**hashedDrops**
Tuning parameters	The parameter ***optional*** determines whether additional clustering is performed to obtain more clusters, and ***extra_cluster*** specifies the number of extra clusters. The parameter ***eu_cut_q*** is used to determine the cut-off between core and non-core cells. The parameter ***md_cut_q*** specifies the cut-off for identifying outlier cells based on the quantile of the Mahalanobis distance.	The *nstarts* and *nsamples* parameters are related to clustering, which does not strongly influence the demultiplexing results, since clustering for most datasets should match the number of hashes. The ***positive.quantile*** parameter is the key factor for determining the cut-offs used to identify singlets.	The parameters ***init.cos.cut*** and ***max.iter*** control the initial values and the number of iterations in EM algorithms, and thus can influence the results. The parameter *converge.threshold* is already set small enough that it does not substantially affect the results. The default parameters ***min.cell.fit*** and *max.cell.fit* provide reasonable settings for determining tags in GLM fitting,	The parameters *min-num-genes* and *min-num-umis* are related to the number of expressed genes and library sizes. Since quality control has already been performed, these two parameters do not strongly influence the results. The key parameters that can be tuned are ***alpha-on-samples*** and ***min-signal-hashtag***, which correspond to the Dirichlet prior	For most low-quality data, the RNA data are of high quality; therefore, the *k.rna* parameter is unlikely to influence the results. But ***k.hto*** can influence the demultiplexing results, since it is the cut-off used to exclude outliers during model fitting. The *tol* parameter is the convergence criterion for the EM algorithm and is already sufficiently small. The following parameters may	Parameters determining the thresholds are: for doublets – ***doublet.nmads***, ***doublet.min***; for singlets – ***confident.nmads***, ***confident.min***.

Data	CMDdemux	HTODemux	deMULTIplex2	demuxEM	demuxmix	hashedDrops
	The parameters ***num_modes*** and ***cut_no*** determine the number of modes in the HTO library size distribution and the cut-off between negatives and doublets. The parameters ***unlabel_cl_cut***, ***unlabel_raw_cut***, and ***unlabel_clr_cut*** are settings for rescuing singlets from unlabelled hashes.		although ***min.cell.fit*** can be adjusted in cases of unlabelled hashes. The parameters ***min.quantile.fit*** and *max.quantile.fit* specify the range of cells used for fitting based on tag count quantiles; their default values are generally suitable for most datasets, though ***min.quantile.fit*** may be adjusted for low-quality data.	concentration parameter on samples and the cut-off between negatives and other categories, respectively.	influence performance: ***pAcpt***, ***alpha***, ***beta***, and ***maxIter***. The ***pAcpt*** parameter determines the cut-off between demultiplexed and undemultiplexed cells, while the other three parameters are related to the EM algorithm. Specifically, ***maxIter*** controls the number of iterations, whereas ***alpha*** and ***beta*** are associated with the threshold distinguishing negatives from other categories.	

Data	CMDdemux	HTODemux	deMULTIplex2	demuxEM	demuxmix	hashedDrops
Treated mouse	Parameter settings: *eu_cut_q* = c(0.93, 0.94, 0.91), *md_cut_q* = 0.93.	Default setting. As the value of *positive.quantile* decreases, more cells are demultiplexed as doublets.	No results were obtained from deMULTIplex2.	Default setting. Changing the two parameters does not significantly influence the demultiplexing.	Parameter settings: *maxIter* = 200, *pAcpt* = 0, *k.hto* = 2. Compared to the default setting, increasing *k.hto* can decrease negatives in the mouse3 cluster, but this effect is limited to a narrow range of *k.hto* values. The parameters *alpha* and *beta* are not used, as the main issue in this dataset is the presence of extra doublets in the mouse1 cluster rather than negatives.	Parameter settings: *doublet.nmads* = 5, *doublet.min* = 5. Increasing *doublet.nmads* and *doublet.min* can decrease the doublet rate in the mouse3 cluster, but this only helps within a limited range of values and does not address the extra negatives in the mouse2 cluster.

Data	CMDdemux	HTODemux	deMULTIplex2	demuxEM	demuxmix	hashedDrops
OT	Parameters settings: *num_modes* = 9, *cut_no* = 10.	Parameter setting: *positive.quantile* = 0.95. The major problem with HTODemux is the presence of extra doublets defined in the OT-G cluster. As the value of the *positive.quantile* parameter decreases, the number of OT-G singlets increases, but false doublets also increase.	Default setting. The performance of deMULTIplex2 on this dataset is already quite good, and further tuning is not necessary.	Default setting. Changing the two parameters does not significantly influence the demultiplexing.	Parameter settings: *maxIter* = 200, *pAcpt* = 0, *k.hto* = 2. Increasing the *k.hto* parameter within a limited range can help increase OT-G singlets. Setting a small value for the *pAcpt* parameter decreases negatives. The *alpha* and *beta* parameters do not influence the results.	Parameter settings: *confident.nmads* = 1, *confident.min* = 1. Since doublets are not the major problem for OT data, the two parameters related to doublets are not tuned. When the parameters *confident.nmads* and *confident.min* are decreased, singlets from all hashtags increase. However, this only works within a limited range of values, and further decreasing the parameters does not improve performance.

Data	CMDdemux	HTODemux	deMULTIplex2	demuxEM	demuxmix	hashedDrops
EMBRYO MULTI-Seq LMO	Parameter settings: *optional* = TRUE, *extra_cluster* = 1, *eu_cut_q* = c(0.9, 0.83, 0.65, 0.87, 0.91, 0.94, 0.8, 0.89, 0.84, 0.53, 0.9, 0.87, 0.88), *md_cut_q* = 0.85, *num_modes* = 10, *cut_no* = 12, *unlabel_cl_cut* = 0.5, *unlabel_raw_cut* = 4.6, *unlabel_clr_cut* = -0.5.	Default setting. As the value of the positive quantile decreases, more cells will be demultiplexed as doublets.	Default setting. Tuning parameters do not significantly change the results.	Cannot get results from demuxEM.	Default setting. Tuning parameters do not significantly change the results.	Parameter settings: *confident.nmads* = 0.5, *confident.min* = 0.5. The major problem of hashedDrops in this dataset is that it produces too many negatives. Reducing *confident.nmads* and *confident.min* can lower the negative rate. However, tuning these parameters only works within a reasonable range; further reduction will no longer improve performance and may increase the risk of misclassifying true negatives as singlets.

Data	CMDdemux	HTODemux	deMULTIplex2	demuxEM	demuxmix	hashedDrops
EMBRYO MULTI-Seq CMO	Parameter settings: *optional* = TRUE, *eu_cut_q* = c(0.9, 0.81, 0.81, 0.79, 0.23, 0.72, 0.95, 0.84, 0.83, 0.9, 0.8, 0.91, 0.79), *label_method* = "expression", *md_cut_q* = 0.51, *num_modes* = 20, *cut_no* = 26.	Default setting. The performance of HTODemux is already good and does not require further tuning.	Default setting. Tuning parameters do not significantly change results.	Cannot get results from demuxEM.	Default setting. The performance of demuxmix is already good and does not require further tuning.	Parameter settings: *confident.nmads* = 1.5, *confident.min* = 1.5. Decreasing these two parameters can slightly improve the singlet rate; however, this only works within a limited range. Continuing to decrease them will misclassify cells in the contaminated cluster as Nxt_460 singlets. At the same time, doublets will also be misclassified as singlets.

Data	CMDdemux	HTODemux	deMULTIplex2	demuxEM	demuxmix	hashedDrops
PDX CellPlex	Parameter settings: *optional* = TRUE, *eu_cut_q* = c(0.98, 0.85, 0.945, 0.93, 0.83), *label_method* = “expression”, *md_cut_q* = 0.52, *num_modes* = 10, *cut_no* = 18.	Parameter setting: *positive.quantile* = 0.95. The performance of HTODemux is reasonable when using default settings. Decreasing this parameter can slightly increase the number of CMO_301 singlets. However, this effect only occurs within a certain range; continuing to decrease the parameter will lead to an increase in doublets.	Default setting. Tuning parameters do not significantly change results.	Cannot get results from demuxEM.	Default settings. Tuning parameters do not significantly change the results. Decreasing the parameter *k.hto* can moderately reduce the doublet rate. However, results without parameter tuning are already similar to those obtained with HTODemux and CMDdemux.	Parameter settings: *confident.nmads* = 0.5, *confident.min* = 0.5. Reducing these two parameters within an appropriate range can increase singlet assignment across all donors.

Data	CMDdemux	HTODemux	deMULTIplex2	demuxEM	demuxmix	hashedDrops
PDX Hashtag Ab	Parameter settings: *optional* = TRUE, *extra_cluster* = 3, *eu_cut_q* = c(0.9, 0.95, 0.95, 0.88, 0.9, 0.88, 0.71), *label_method* = “expression”, *md_cut_q* = 0.4, *num_modes* = 8, *cut_no* = 4.	Parameter setting: *positive.quantile* = 0.95. Decreasing the parameter within a reasonable range can increase singlets. However, further decreasing the parameter will increase doublets as well.	Default setting. Tuning parameters even makes the results worse, which reassigns most negatives as HTO_1 singlets and singlets from other donors as doublets.	Cannot get results from demuxEM.	Default setting. Tuning parameters do not change the results much. Moreover, the results without parameter tuning are already similar to those of HTODemux.	Parameter settings: *confident.nmads* = 0.1, *confident.min* = 0.1, *doublet.nmads* = 0.5, *doublet.min* = 0.5. Decreasing the four parameters within an appropriate range can significantly increase both singlets and doublets. However, further decreasing *confident.nmads* and *confident.min* does not continuously increase singlets, while further decreasing *doublet.nmads* and *doublet.min* carries the risk of over-labelling doublets.

Data	CMDdemux	HTODemux	deMULTIplex2	demuxEM	demuxmix	hashedDrops
PDX<br>Multi-Seq<br>CMO	Parameter settings: *eu_cut_q* = c(0.95, 0.95, 0.81, 0.86), *md_cut_q* = 0.8, *num_modes* = 7, *cut_no* = 4.	Parameter setting: *positive.quantile* = 0.95. Decreasing the parameter within a reasonable range can increase singlets. However, continuing to decrease the parameter will increase doublets as well.	Default setting. Tuning parameters do not significantly change results.	Cannot get results from demuxEM.	Parameter settings: *pAcpt* = 0.1, *k.hto* = 1. Decreasing the two parameters within a reasonable range will assign more Nxt_451 and Nxt_452 singlets, but continuously decreasing them will not further improve its performance.	Parameter settings: *confident.nmads* = 0.5, *confident.min* = 0.5, *doublet.nmads* = 1, *doublet.min* = 1. Decreasing the four parameters within an appropriate range can significantly increase both singlets and doublets. However, continuously decreasing *confident.nmads* and *confident.min* will not further increase singlets, and continuously decreasing *doublet.nmads* and *doublet.min* carries the risk of incorrectly labelling doublets.

Since the benchmarking results for PBMC data do not show notable differences, they are not provided here.

GMM-Demux in its basic usage does not require parameter tuning; therefore, we do not include it in this table.

The BFF methods are implemented within a function called *GenerateCellHashingCalls*. This function focuses on obtaining consensus calls from different demultiplexing methods, so its parameters are related to consensus thresholds rather than specific BFF algorithm parameters. Therefore, we do not include BFF in this table.

Possible tuned parameters are shown in bold.

References

[1] Marlon Stoeckius, Shiwei Zheng, Brian Houck-Loomis, Stephanie Hao, Bertrand Z Yeung, William M Mauck, Peter Smibert, and Rahul Satija. Cell hashing with barcoded antibodies enables multiplexing and doublet detection for single cell genomics. *Genome biology*, 19:1–12, 2018.

[2] Jonathan A Griffiths, Arianne C Richard, Karsten Bach, Aaron TL Lun, and John C Marioni. Detection and removal of barcode swapping in single-cell rna-seq data. *Nature communications*, 9(1):1–6, 2018.

[3] Jellert T Gaublomme, Bo Li, Cristin McCabe, Abigail Knecht, Yiming Yang, Eugene Drokhlyansky, Nicholas Van Wittenberghe, Julia Waldman, Danielle Dionne, Lan Nguyen, et al. Nuclei multiplexing with barcoded antibodies for single-nucleus genomics. *Nature communications*, 10(1):2907, 2019.

[4] Hongyi Xin, Qiuyu Lian, Yale Jiang, Jiadi Luo, Xinjun Wang, Carla Erb, Zhongli Xu, Xiaoyi Zhang, Elisa Heidrich-O'Hare, Qi Yan, et al. Gmm-demux: sample demultiplexing, multiplet detection, experiment planning, and novel cell-type verification in single cell sequencing. *Genome biology*, 21:1–35, 2020.

[5] Gregory J Boggy, GW McElfresh, Eisa Mahyari, Abigail B Ventura, Scott G Hansen, Louis J Picker, and Benjamin N Bimber. Bff and cellhashr: analysis tools for accurate demultiplexing of cell hashing data. *Bioinformatics*, 38(10):2791–2801, 2022.

[6] Hans-Ulrich Klein. demuxmix: demultiplexing oligonucleotide-barcoded single-cell rna sequencing data with regression mixture models. *Bioinformatics*, 39(8):btad481, 2023.

[7] Qin Zhu, Daniel N Conrad, and Zev J Gartner. demultiplex2: robust sample demultiplexing for scrna-seq. *Genome Biology*, 25(1):37, 2024.

[8] George Howitt, Yuzhou Feng, Lucas Tobar, Dane Vassiliadis, Peter Hickey, Mark A Dawson, Sarath Ranganathan, Shivanthan Shanthikumar, Melanie Neeland, Jovana Maksimovic, et al. Benchmarking single-cell hashtag oligo demultiplexing methods. *NAR Genomics and Bioinformatics*, 5(4):lqad086, 2023.

[9] Balaji Virassamy, Franco Caramia, Peter Savas, Sneha Sant, Jianan Wang, Susan N Christo, Ann Byrne, Kylie Clarke, Emmaline Brown, Zhi Ling Teo, et al. Intratumoral cd8+ t cells with a tissue-resident memory phenotype mediate local immunity and immune checkpoint responses in breast cancer. *Cancer Cell*, 41(3):585–601, 2023.

[10] Ariel A Hippen, Dalia K Omran, Lukas M Weber, Euihye Jung, Ronny Drapkin, Jennifer A Doherty, Stephanie C Hicks, and Casey S Greene. Performance of computational algorithms to deconvolve het-

erogeneous bulk ovarian tumor tissue depends on experimental factors. *Genome biology*, 24(1):239, 2023.

[11] Daniel V Brown, Casey JA Anttila, Ling Ling, Patrick Grave, Tracey M Baldwin, Ryan Munnings, Anthony J Farchione, Vanessa L Bryant, Amelia Dunstone, Christine Biben, et al. A risk-reward examination of sample multiplexing reagents for single cell rna-seq. *Genomics*, 116(2):110793, 2024.

Supplementary Figures

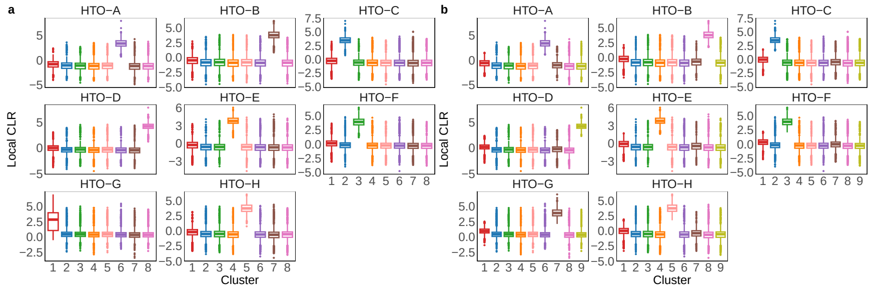

Fig. S1: **Hashtag expression across clusters for different values of k.** Hashtag expression is represented by the local CLR value. (a) k = 8. (b) k = 9.

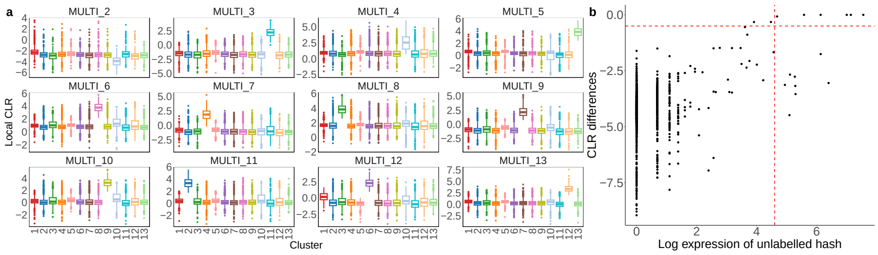

Fig. S2: **The unlabelled hashtag in the EMBRYO LMO data.** (a) Local CLR expression of each hashtag across clusters. (b) Scatter plot of all temporary negatives. The x-axis represents the log expression of MULTI_2, and the y-axis represents the difference between the CLR value of the unlabelled hashtag (MULTI_2) and the maximum CLR value in each cell. In theory, true droplets associated with the unlabelled hashtag should exhibit high expression of the unlabelled hashtag, and the CLR difference should be close to zero. Two red lines represent the cut-offs: the cut-off for the CLR difference is set at -0.5, and the cut-off for the log expression of the unlabelled hashtag is set at 4.6. The six droplets located in the top right region of the scatter plot are rescued and reclassified as MULTI_2 singlets.

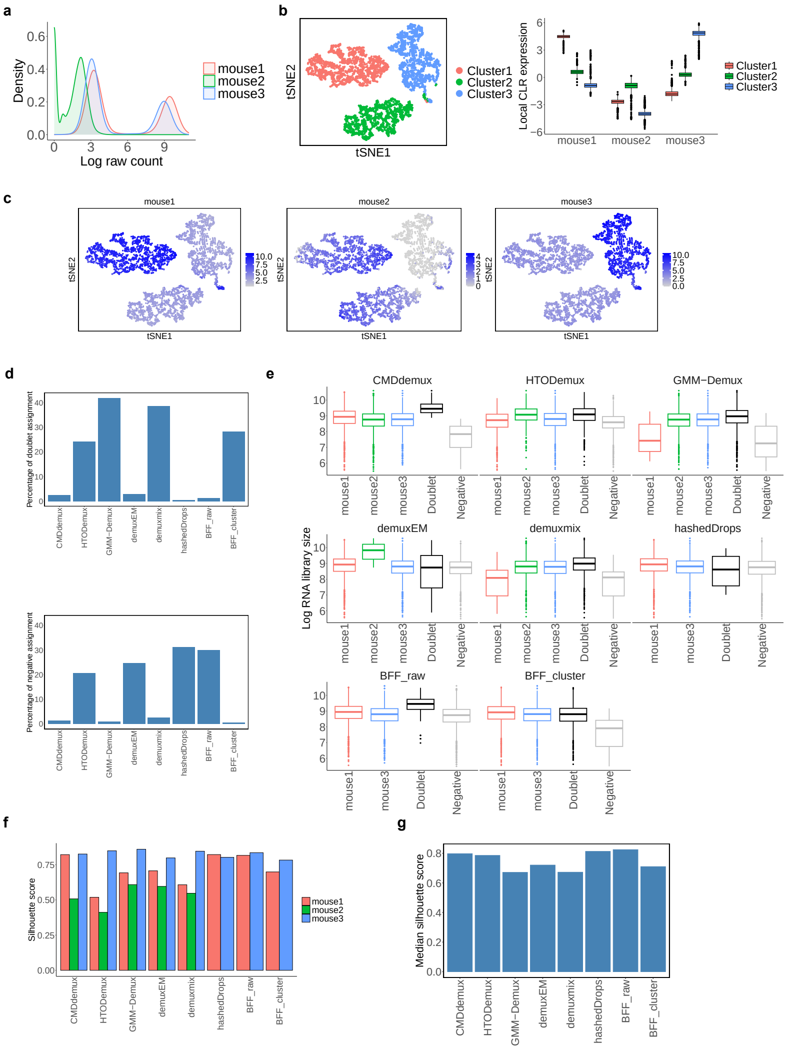

Fig. S3: **Data quality and CMDdemux performance on the treated mouse dataset.** (a) Distribution of log-transformed hash counts. (b) t-SNE plot showing three clusters and the expression of each hashtag in each cluster. Mouse1, mouse2, and mouse3 are highly expressed in clusters 1, 2, and 3, respectively. (c) t-SNE plots colored by log-transformed raw hash counts of the corresponding mouse hashtags. Except for mouse2, the other two hashtags show strong expression in two distinct clusters. (d) Proportion of doublets and negatives among all droplets. (e) Distribution of mRNA library sizes across assignment categories from different methods. (f) Median silhouette scores for each demultiplexing category by different methods. (g) Median silhouette score across all singlets.

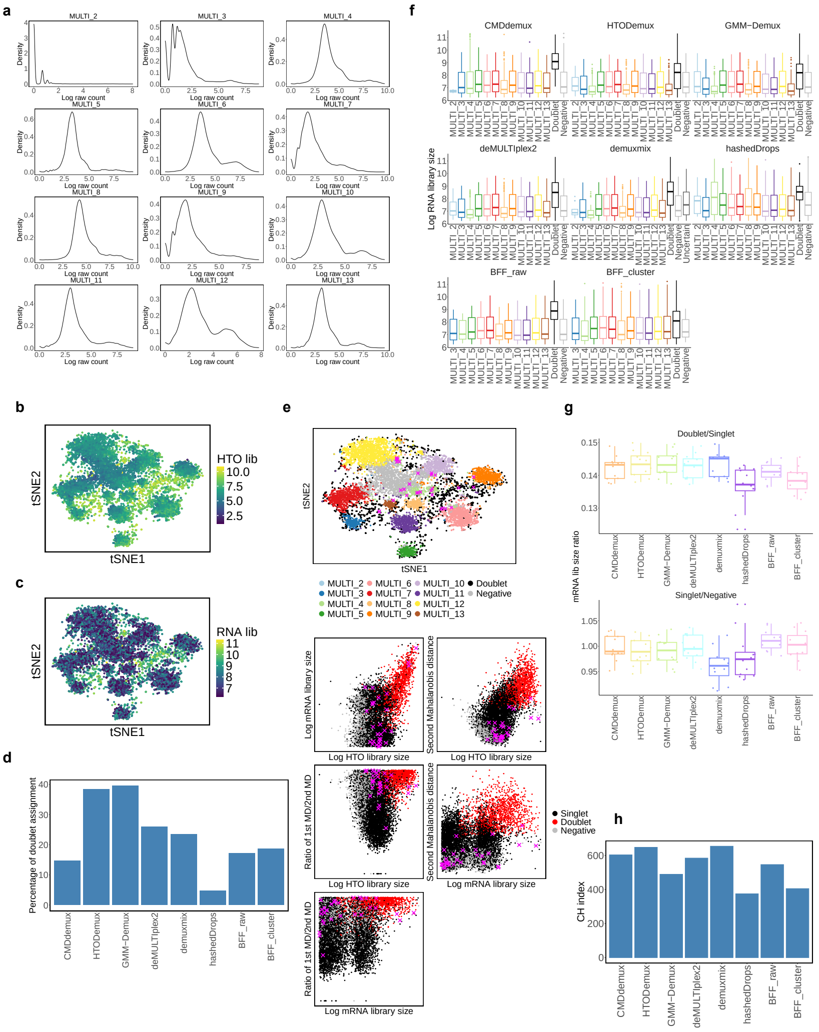

Fig. S4: **Quality of the EMBRYO LMO data and performance of CMDdemux.** (a) Each plot shows the distribution of log counts for the corresponding hashtag. (b) t-SNE plot colored by HTO library sizes. (c) t-SNE plot colored by mRNA library sizes. (d) Percentage of doublets assigned by different methods. (e) Top panel: randomly selected CMDdemux-defined extra_MULTI_4 singlets shown as magenta crosses. Bottom panel: assessment of these extra_MULTI_4 singlets using the "CheckAssign2DPlot" function in CMDdemux. (f) Distribution of mRNA library sizes for different demultiplexing categories across methods. (g) Top panel: ratio of the median mRNA library size in doublets to that of singlets in each singlet category. Bottom panel: ratio of the median mRNA library size in each singlet category to that of all negatives. Each dot in the boxplot represents a singlet category. (h) CH index for all singlet classification results across different methods.

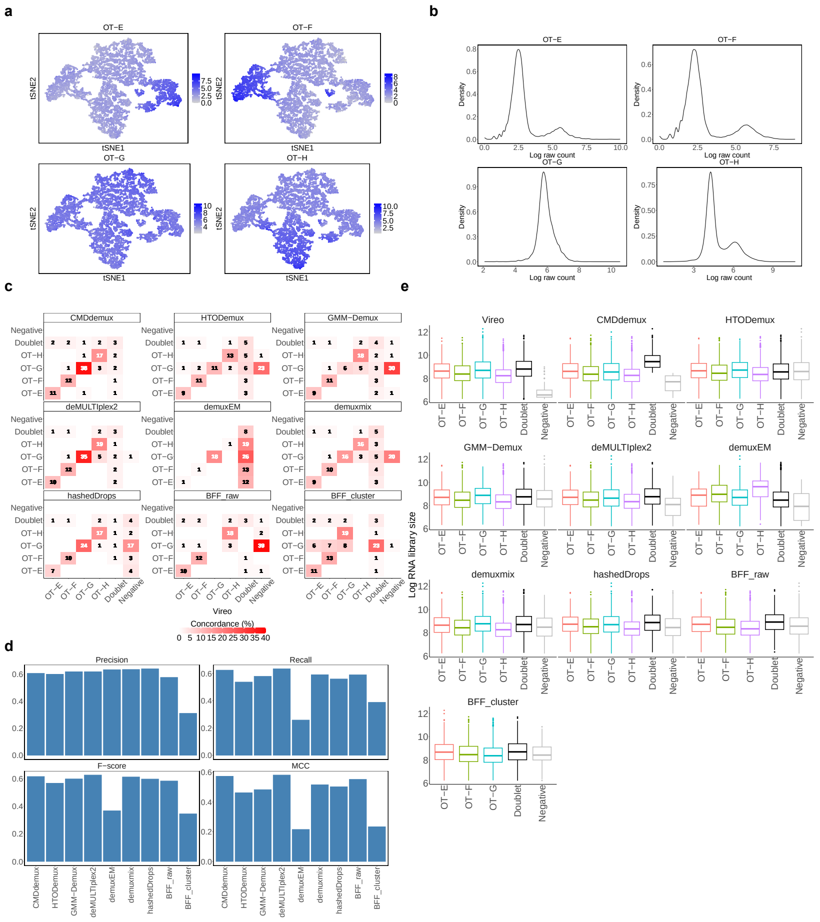

Fig. S5: **Quality of OT data and performance of CMDdemux.** (a) tSNE plot colored by the log-transformed raw count expression of each hashtag. (b) Distribution of log-transformed raw counts for each hashtag. (c) Concordance of demultiplexing results from different methods with Vireo, shown for each category. (d) Performance evaluation of different methods based on average precision, recall, F-score, and MCC. (e) Boxplot of mRNA library sizes across different demultiplexing categories assigned by various methods.

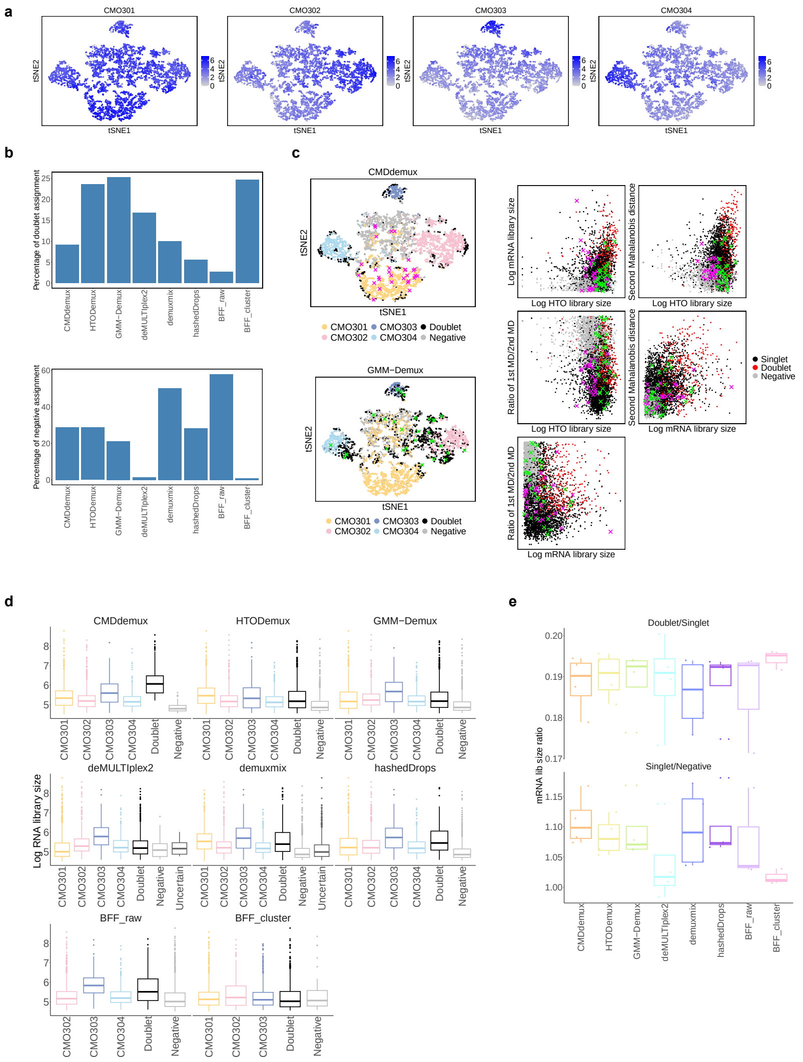

Fig. S6: **Quality of the PDX CellPlex data and performance of CMDdemux.** (a) t-SNE plot colored by the raw expression of each hashtag. (b) Percentage of doublets and negatives assigned by different methods. (c) Extra CMO301 singlets are shown as magenta crosses in the t-SNE plot colored by CMDdemux demultiplexing, and extra doublets are shown as green crosses in the t-SNE plot colored by GMM-Demux demultiplexing. The right panel was generated using the CheckAssign2DPlot function from CMDdemux. (d) mRNA library sizes for different demultiplexing categories across various methods. (e) Median mRNA library size ratios between doublets and singlets, and between singlets and negatives. Each dot in the boxplot represents the median mRNA library size of a singlet category and its relative ratio to the median mRNA library sizes across all doublets or negatives.

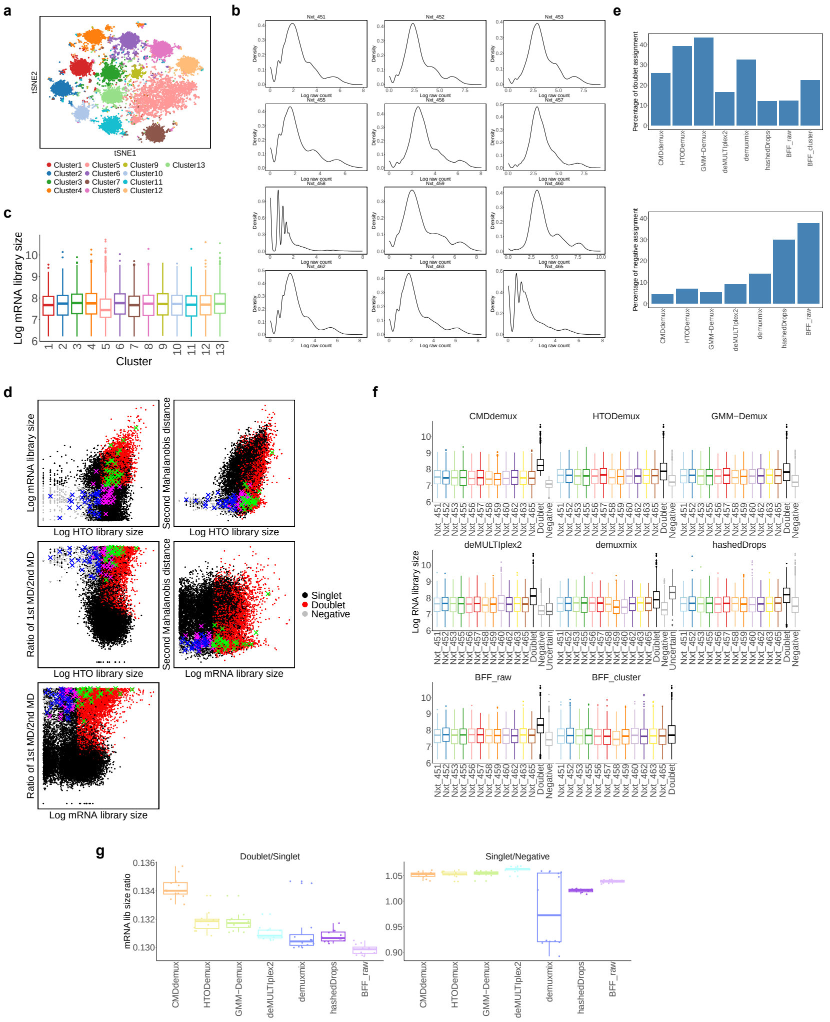

Fig. S7: **Quality of the EMBRYO CMO dataset and performance of CMDdemux.** (a) tSNE plot colored by cluster assignments. (b) Distribution of log-transformed raw counts of each hashtag. (c) Log-transformed mRNA library sizes for each cluster. (d) Examination of demultiplexing results in the contaminated cluster. The plot is generated by the CheckAssign2DPlot function in CMDdemux. Thirty randomly selected singlets, doublets, and negatives are shown in magenta, green, and blue crosses, respectively. (e) Percentage of doublets (top panel) and negatives (bottom panel) identified by different methods. (f) mRNA library sizes across different demultiplexing categories from each method. (g) Ratio of median mRNA library sizes: doublets to singlets (left panel) and singlets to negatives (right panel). In the left panel, each dot represents the ratio of the median library size of all doublets to that of singlets in a specific singlet category. In the right panel, each dot represents the ratio of the median library size of singlets (in a specific category) to that of all negatives.

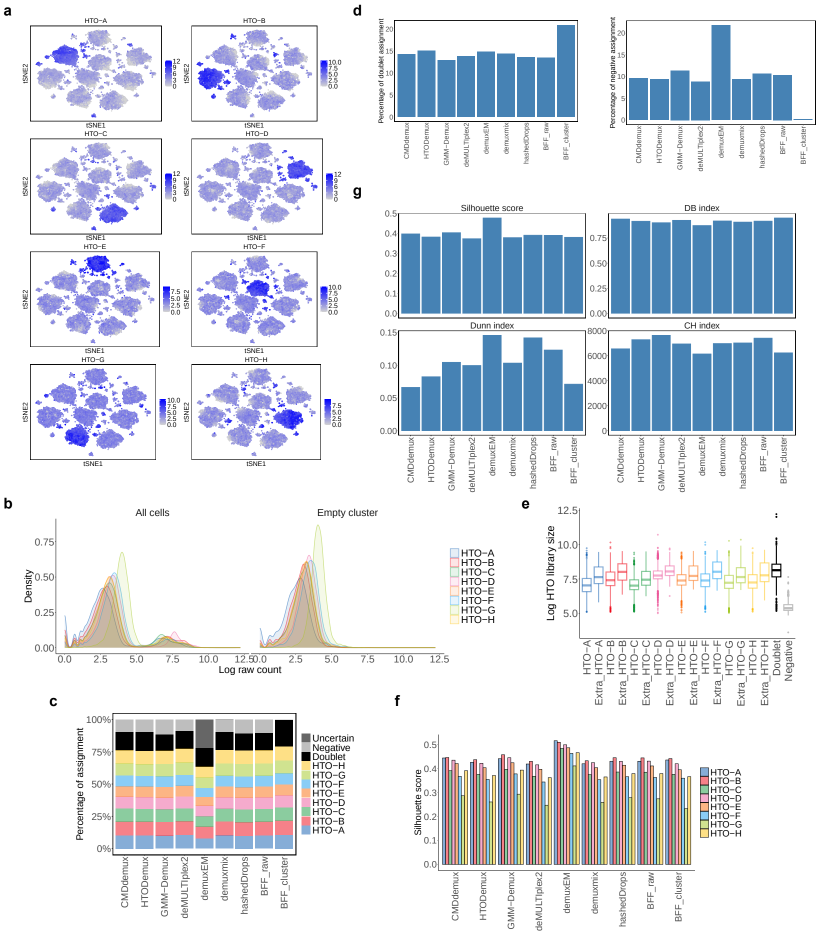

Fig. S8: **The quality of PBMC data and the performance of CMDdemux.** (a) t-SNE plot colored by the log-scaled expression of raw hash counts. (b) Distribution of log-scaled hash counts across all cells (left panel) and within the empty cluster (right panel). (c) Proportion of cells assigned to different categories by various demultiplexing methods. (d) Percentages of doublets (left panel) and negatives (right panel) identified by different methods. (e) Log-scaled HTO library sizes of cells in different demultiplexing categories defined by CMDdemux. Extra singlets refer to cells identified as singlets by CMDdemux but as doublets by HTODemux. (f) Median silhouette score of each singlet category across different methods. (g) Demultiplexing performance for singlets across different methods, evaluated using the silhouette score, Davies–Bouldin (DB) index, Dunn index, and Calinski–Harabasz (CH) index. The median silhouette score across all singlets is shown in the plot.

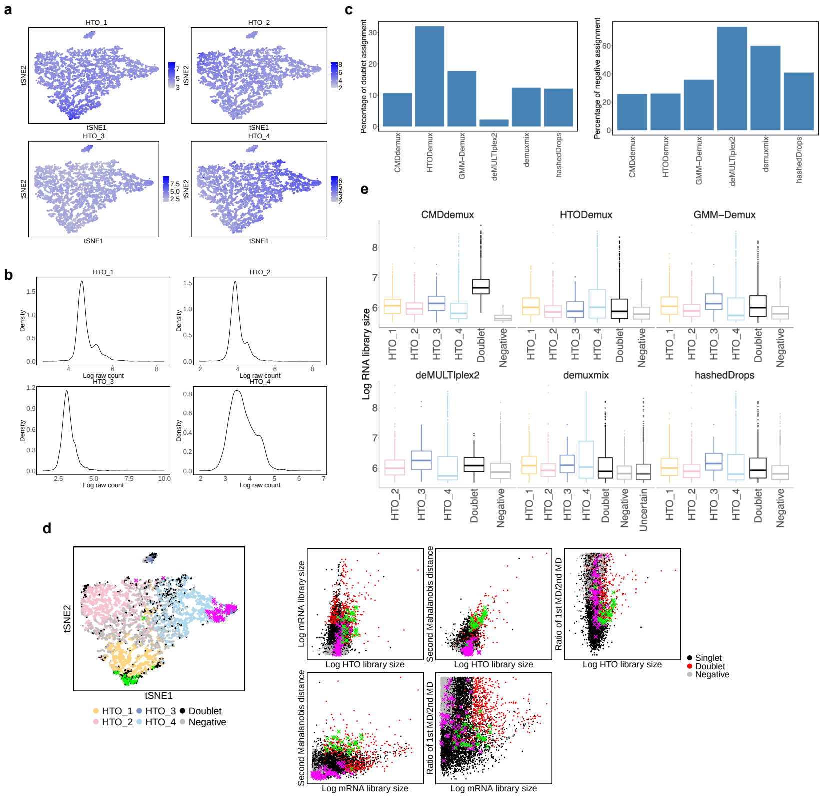

Fig. S9: **Quality of PDX Hashtag Ab data and performance of CMDdemux.** (a) t-SNE plot colored by the raw counts of each hashtag on a log scale. (b) Distribution of raw counts for each hashtag on a log scale. (c) Percentage of doublet assignments (left panel) and negative assignments (right panel) from different methods. (d) t-SNE plot colored by CMDdemux demultiplexing results. Magenta crosses indicate cells assigned as HTO_3 or HTO_4 singlets by CMDdemux but as doublets by both HTODemux, GMM-Demux and hashedDrops. Green crosses indicate cells assigned as doublets by CMDdemux but as HTO_1 singlets by HTODemux, GMM-Demux and hashedDrops. Thirty extra HTO_3 singlets, 30 extra HTO_4 singlets, and 30 extra doublets assigned by CMDdemux are randomly selected and shown in the left panel, with their locations indicated in the right panel, which is generated using the CheckAssign2DPlot function in CMDdemux. (e) mRNA library sizes of cells assigned to different demultiplexing categories by various methods.

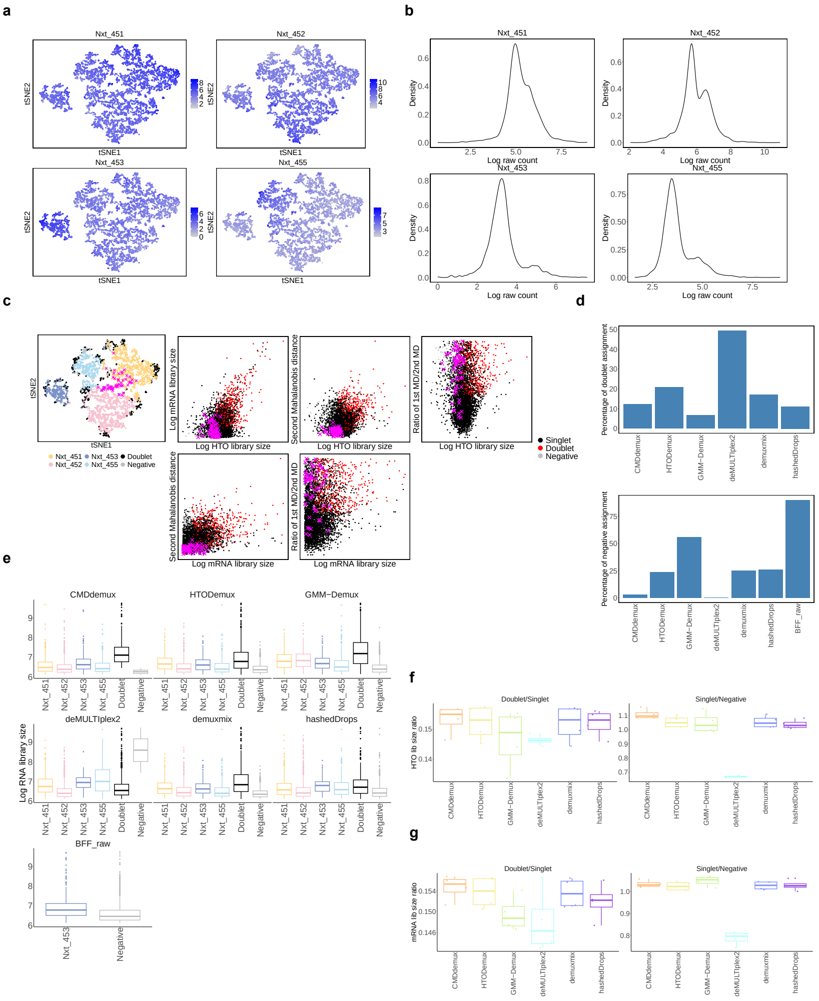

Fig. S10: **Quality of PDX MULTI-Seq CMO data and performance of CMDdemux.** (a) t-SNE plot colored by the log-transformed expression of raw hashtag counts. (b) Distribution of raw hashtag counts on a log scale. (c) t-SNE plots showing CMDdemux results. In the left panel, CMDdemux assignments are displayed, with 30 randomly selected extra Nxt_451 and 30 extra Nxt_452 singlets (classified as negatives by HTODemux, demuxmix and hashedDrops) marked by magenta crosses. The right panel shows the CheckAssign2DPlot output from CMDdemux. (d) Proportions of doublets (top) and negatives (bottom) assigned by different demultiplexing methods. (e) Log-transformed mRNA library sizes of cells assigned to different categories by various methods. (f) Left: Ratio of median HTO library size of doublets to that of each singlet category. Right: Ratio of median HTO library size of each singlet category to that of negatives. Each dot represents a singlet category. (g) Left: Ratio of median mRNA library size of doublets to that of each singlet category. Right: Ratio of median mRNA library size of each singlet category to that of negatives. Each dot represents a singlet category.

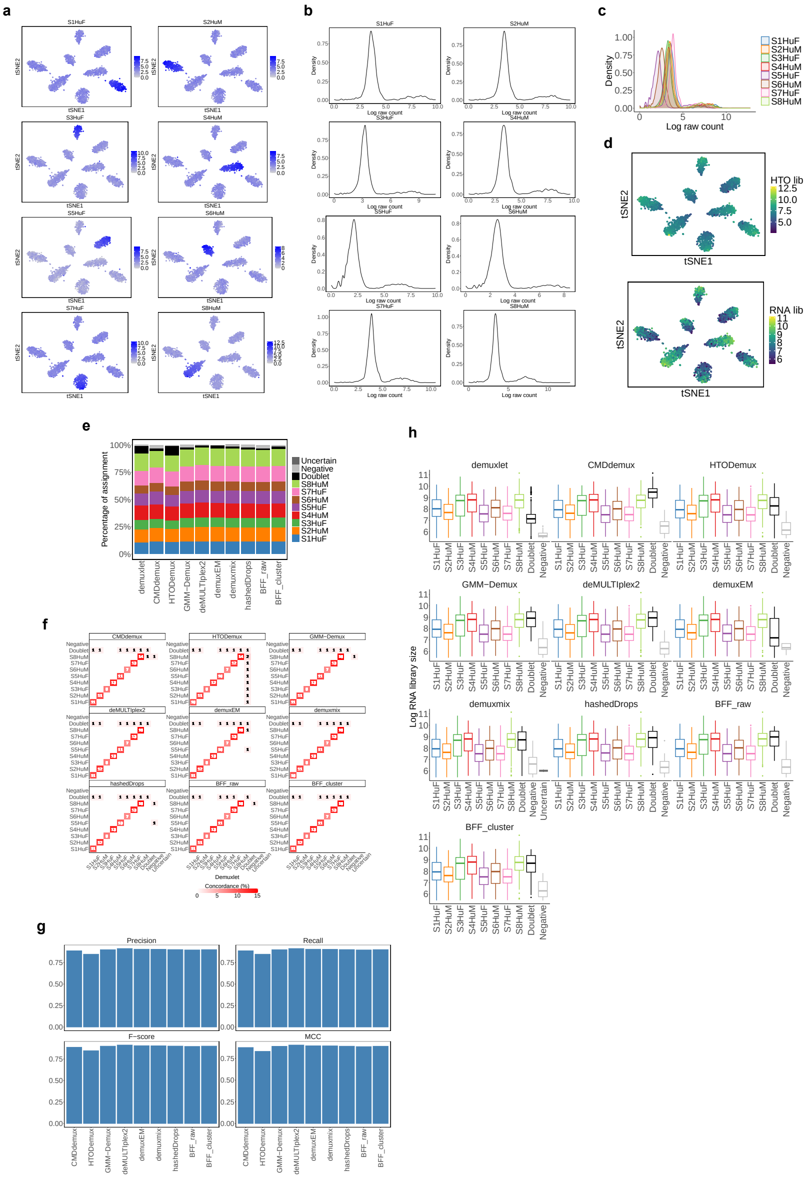

Fig. S11: **Quality of the human brain dataset and performance of CMDdemux.** (a) t-SNE plot colored by the raw expression (log scale) of each hashtag. (b) Distribution of raw expression values (log scale) for each hashtag. (c) Distribution of raw hashtag counts (log scale), combined across different hashtags. (d) t-SNE plots colored by HTO library sizes (upper panel) and mRNA library sizes (lower panel). (e) Proportion of cells assigned to each donor by different methods. (f) Concordance with demuxlet for each demultiplexing category, across different methods. (g) Average precision, recall, F-score, and MCC across all demultiplexing categories for each method. (h) mRNA library sizes across demultiplexing categories for each method.

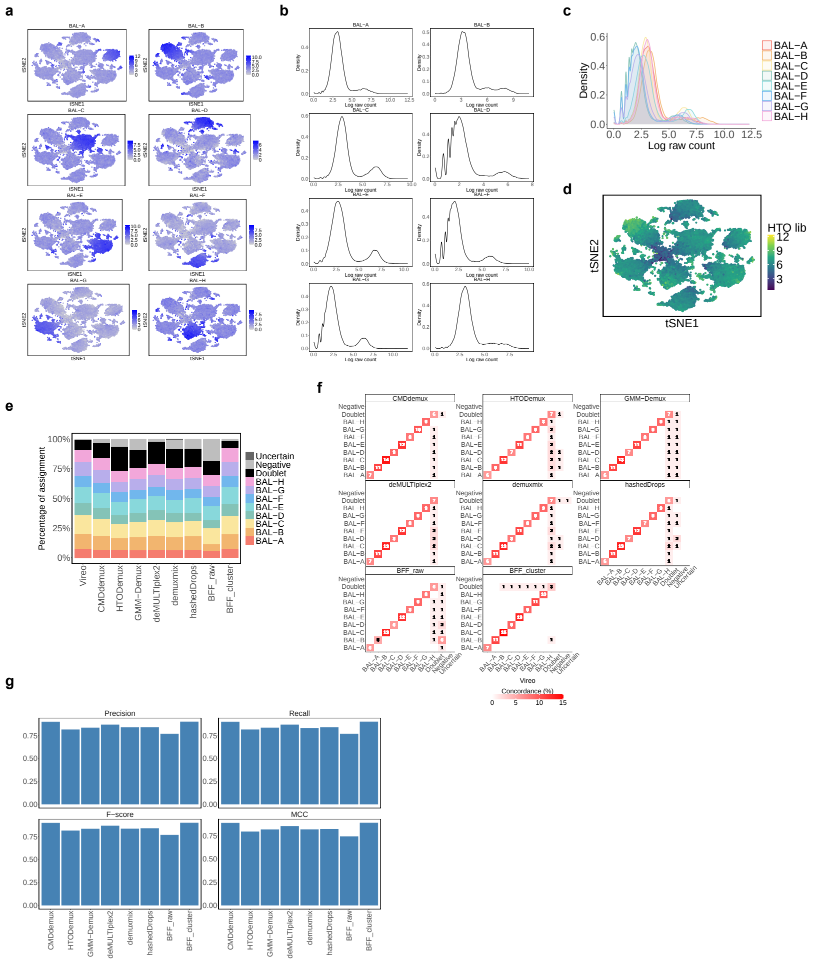

Fig. S12: **Quality of the BAL dataset and performance of CMDdemux.** Gene expression data is not available for this dataset. (a) t-SNE plot colored by the raw expression (log scale) of each hashtag. (b) Distribution of raw expression (log scale) for each hashtag. (c) Distribution of raw hashtag counts (log scale). (d) t-SNE plot colored by HTO library sizes. (e) Proportion of cells assigned to each donor by different methods. (f) Concordance with Vireo for each demultiplexing category, across different methods. (g) Average precision, recall, F-score, and MCC across all demultiplexing categories for each method.

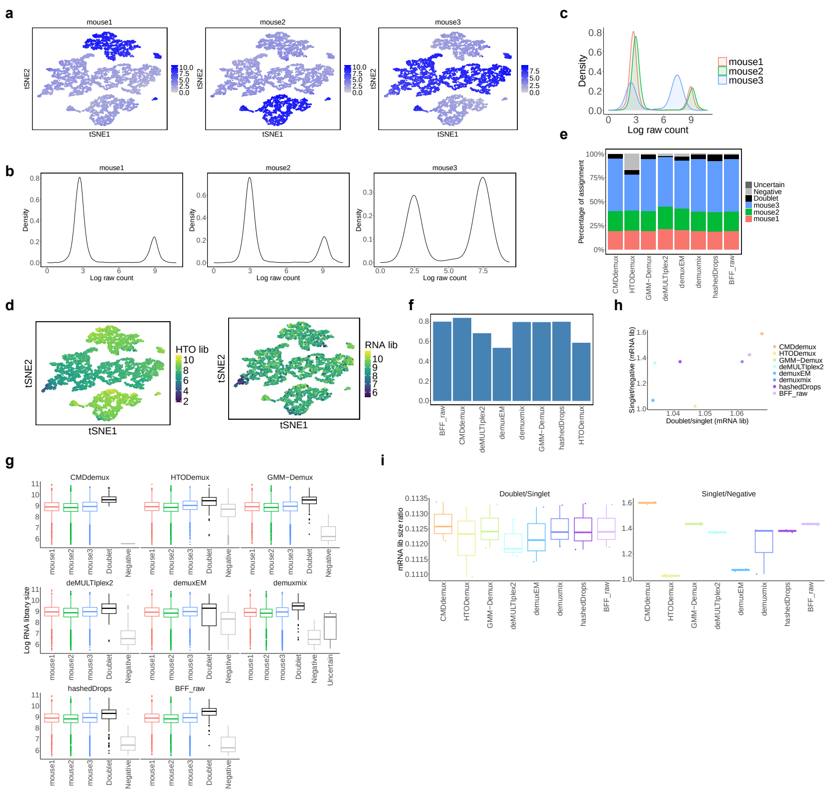

Fig. S13: **Quality of the vehicle mouse dataset and performance of CMDdemux.** (a) t-SNE plot colored by the raw expression (log scale) of each hashtag. (b) Distribution of raw expression (log scale) for each hashtag. (c) Distribution of raw hashtag counts (log scale). (d) t-SNE plot colored by HTO library sizes (left panel) and mRNA library sizes (right panel). (e) Proportion of cells assigned to each donor. (f) Median silhouette score across all singlet categories. (g) mRNA library sizes for each demultiplexing category, across different methods. (h) Ratio of the median mRNA library size of doublets to singlets (from the singlet category with the highest median mRNA library size), and of singlets (from the singlet category with the lowest median) to negatives. (i) Left panel: ratio of the median mRNA library size of doublets to singlets. Right panel: ratio of singlets to negatives. Each dot represents one singlet category.
